# Robot-Assisted Radical Prostatectomy Associated with Decreased Persistent Postoperative Opioid Use

**DOI:** 10.1089/end.2019.0788

**Published:** 2020-04-16

**Authors:** Eugene Shkolyar, I-Fan Shih, Yanli Li, Jaime A. Wong, Joseph C. Liao

**Affiliations:** ^1^ Department of Urology, Stanford University School of Medicine, Stanford, California, USA.; ^2^ VA Palo Alto Health Care System, Palo Alto, California, USA.; ^3^ Intuitive Surgical, Inc., Sunnyvale, California, USA.

**Keywords:** prostatic neoplasms, prostatectomy, robotic surgical procedures, analgesics, opioid

## Abstract

***Introduction:*** Minimally invasive surgery offers reduced pain and opioid use postoperatively compared with open surgery, but large-scale comparative studies are lacking. We assessed the incidence of persistent opioid use after open and robot-assisted radical prostatectomy (RARP).

***Materials and Methods:*** We performed a retrospective claims database cohort study of opioid-naive (i.e., no opioid prescriptions 30–180 days before index surgery) adult males who underwent radical prostatectomy for prostate cancer from July 2013 to June 2017. For patients who filled a perioperative opioid prescription (30 days before to 14 days after surgery), we calculated the incidence of new persistent postoperative opioid use (≥1 prescription 90–180 days after surgery). Multivariable logistic regression was performed to investigate the association between the surgical approach, patient risk factors, and persistent opioid use.

***Results:*** Twelve thousand two hundred seventy-eight radical prostatectomy patients filled an opioid prescription perioperatively (1510 [12%] open and 10,768 [88%] robot assisted). Of these, 846 (6.9%) patients continued to fill opioid prescription(s) 90 to 180 days after surgery. Patients undergoing RARP were 35% less likely to develop new persistent opioid use compared with those undergoing open radical prostatectomy (6.5% *vs* 9.7%; adjusted odds ratio 0.65; 95% confidence interval 0.54, 0.79). Other independent risk factors included living in the southern, western, or north central United States; preoperative comorbidity; and tobacco use.

***Conclusions:*** Approximately 6.9% of opioid-naive patients continued to fill opioid prescriptions 90 days after radical prostatectomy. The risk of persistent opioid use was significantly lower among patients undergoing a robot-assisted *vs* open approach. Further efforts are needed to develop postoperative opioid prescription protocols for patients undergoing radical prostatectomy.

## Introduction

Over the past two decades, the United States has seen a sharp rise in opioid medication prescriptions. This rise has coincided with an increase in drug overdoses, with prescription opioids accounting for more than 17,000 overdose deaths in 2017.^1^ Opioids have long played a critical role in pain management among surgical patients, and for many patients, surgery may be their first opioid exposure.^2^ Among previously opioid-naïve patients, ∼6% continue to use opioids more than 3 months after surgical procedures.^3^ In one study, over 80% of patients undergoing low-risk surgery received a prescription for opioid medications, and those who were prescribed perioperative opioids were 44% more likely to receive opioids 1 year postoperatively compared with those who did not receive a perioperative prescription.^4,5^ Additionally, the majority of opioids prescribed in the perioperative setting go unused and few are appropriately disposed of, thus misuse and diversion represent a public health challenge.^6^ Understanding the factors that place patients at higher risk for persistent or prolonged opioid use is critical to minimizing opioid dependence after surgery.^5^

Chronic opioid use represents a significant burden on patients and the health care system. Opioid abuse leads to an estimated additional $14,810 in per-patient annual health care costs due to higher health care utilization.^7^ In patients undergoing plastic and reconstructive surgery, risks for persistent (between 90 and 180 days after surgery) and prolonged (more than 180 days after surgery) use include being prescribed an opioid in the perioperative setting, procedure type, number of medical comorbidities, and history of mental health disorders or substance abuse.^8^ Although work has been done to characterize rates of perioperative opioid use after radical prostatectomy, data on persistent and prolonged opioid use are limited.^9–12^

Robot-assisted radical prostatectomy (RARP) accounts for 85% of radical prostatectomies performed in the United States.^13^ The remaining 15% are primarily open radical prostatectomies (ORPs), with a small minority performed laparoscopically or using a perineal approach. When compared with ORP, RARP is associated with decreased operative time, blood loss, length of hospital stay, and pain in the week after surgery.^14^ These benefits have led to the widespread adoption of RARP and incorporation of robot-assisted surgery into Enhanced Recovery After Surgery care pathways that focus on early return to preoperative functional status.^15^ It is unknown, however, whether the benefits of RARP with regard to early postoperative pain affect long-term opioid use.

Using population-based insurance claims data, we aimed to evaluate the incidence of new persistent and prolonged opioid use among patients undergoing radical prostatectomy in the United States. In addition, we evaluated the impact of initial opioid prescribing patterns in the perioperative period, patient demographics, and surgical approach (ORP *vs* RARP) on long-term opioid use.

## Materials and Methods

### Data source and study sample

We analyzed the IBM^®^ MarketScan^®^ Research Databases (MarketScan), which include data on inpatient, outpatient, and pharmacy services from a range of employer-provided health insurance plans.^16^ MarketScan claims databases contain health services data for more than 40 million employees, dependents, and retirees in the United States. As these are aggregated, deidentified Health Insurance Portability and Accountability Act (HIPAA)-compliant databases, informed consent and institutional review board approval were not required for our analysis.

The study population comprised male patients who were aged 18 years or older and underwent inpatient radical prostatectomy for prostate cancer between July 1, 2013, and June 30, 2017. Patients were additionally required to be continuously enrolled with medical and prescription drug coverage, from 180 days before to 180 days after surgery. As a proxy to identify opioid-naïve patients, we excluded patients whose hospital length of stay was greater than 10 days, those who were diagnosed with opioid dependence or abuse (*International Classification of Diseases and Related Health Problems, Ninth Revision* [ICD-9] code 305.x or 304.x), and those who had filled at least one opioid prescription in the 31 to 180 days before their surgery. Patients who did not fill an opioid prescription in the perioperative period (30 days before prostatectomy to 14 days after discharge) were excluded. Patients who underwent laparoscopic prostatectomy were also excluded due to the relative rarity of this approach. ICD-9 and *International Classification of Diseases and Related Health Problems*, *10th Revision* (ICD-10), Current Procedural Terminology (CPT) codes and hospital billing information were used to define the eligible prostatectomy cases and differentiate surgical approaches (Supplementary Table S1).

### Outcome variables

**FIG. 1.**
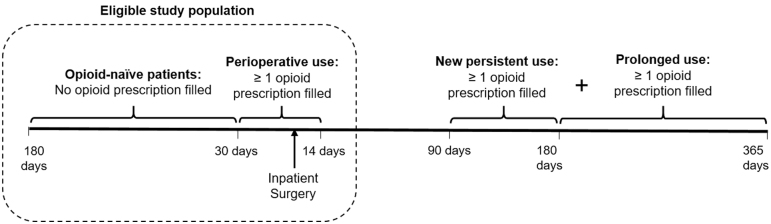
Study criteria and outcome definition. Patients were included if they did not fill an opioid prescription 180 days before the surgery (opioid-naïve) and had at least one opioid prescription filled 30 days before surgery to 14 days after discharge (perioperative use). The outcome of new persistent opioid use was defined as at least one opioid prescription filled between 90 and 180 days after the discharge; prolonged use was defined as one or more additional opioid prescription(s) filled between 180 and 365 days after discharge.

The primary outcome was new persistent opioid use, defined as an opioid prescription fulfillment between 90 and 180 days after surgery, among patients who filled an opioid in the 30 days before prostatectomy or within 14 days after discharge (i.e., perioperatively) (Fig. 1). This definition of new persistent opioid use is consistent with prior studies and represents a time frame in which routine postoperative recovery would be expected following radical prostatectomy.^3,8^ Pharmacy data were collected for 180 days after discharge to determine whether and when opioid prescriptions were filled as well as the duration and types of opioids filled. Dosages for each type of opioid medication were converted into oral morphine equivalents (OMEs).

### Patient factors

We assessed patients' baseline sociodemographic and clinical characteristics, including age, sex, insurance plan, year of surgery, and Charlson Comorbidity Index (CCI) scores. Insurance plans were classified into preferred provider organization, comprehensive insurance, health maintenance organization, point of service, and other insurance plans. We also identified conditions known to increase the risk of opioid overuse based on existing literature, including obesity, current or previous tobacco use, alcohol abuse, drug abuse, and mental health disorders (psychosis or depression), using the ICD-9/ICD-10 diagnosis codes.^3,8,9^ Patient comorbidities and the above risk factors were evaluated at the index hospitalization and in the 180-day preoperative period.

### Statistical analyses

Descriptive statistics were calculated for demographic variables, comorbidities, and opioid utilization among patients undergoing ORP and RARP. Adjusted odds ratios (aORs) were calculated using multivariable logistic regression to investigate the association between surgical approach and new persistent opioid use after controlling for sociodemographic and clinical covariates. aORs were considered statistically significant if the 95% confidence interval (CI) did not cross one. In a sensitivity analysis, we calculated the rates and risk factors of prolonged opioid use—defined as additional opioid prescription fulfillment 181 to 365 days after discharge in patients with persistent (90–180 days after discharge) opioid use—among participants who were continuously enrolled for 365 or more days after discharge.

We further conducted propensity score matches (PSM) between ORP and RARP to minimize potential bias from differences in baseline characteristics. We used multivariable logistic regression to calculate the propensity score accounting for all covariates. The greedy matching algorithm was used to generate the matched study samples at 1:1 ratio. We assessed the covariate balance achieved by the PSM using standardized differences. A threshold of less than 0.1 was assumed to indicate a negligible difference in baseline characteristics between the two groups. All analyses were performed using SAS software, version 9.4 (SAS Institute, Inc., Cary, NC).

## Results

**FIG. 2.**
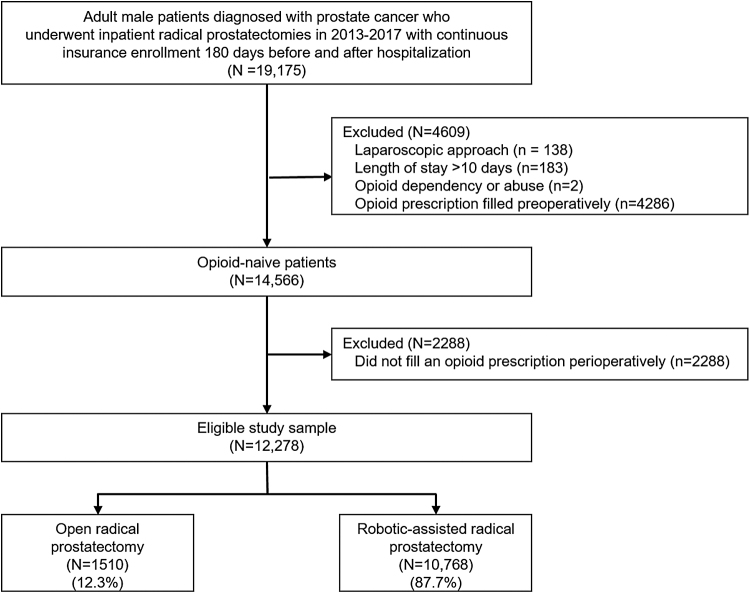
Study flow.

We identified 19,175 continuously enrolled adult males who underwent inpatient radical prostatectomy for prostate cancer between July 2013 and June 2017. Among the 14,566 opioid-naïve patients identified, 15.7% (2288) did not fill an opioid prescription perioperatively. After applying exclusion criteria, the final cohort consisted of 12,278 patients: 1510 (12.3%) underwent ORP and 10,768 (87.7%) underwent RARP (Fig. 2). Descriptive data stratified by surgical approach are summarized in Table 1. Patients who underwent ORP were older, had more comorbidities, and a greater proportion lived in the southern region of the United States compared with those who underwent RARP.

**Table 1.** Demographic and Preoperative Patient Characteristics

	ORP (*n* = 1510),* n *(%)	RARP (*n* = 10,768),* n *(%)
Age, years		
18–54	261 (17.3)	2185 (20.3)
55–65	914 (60.5)	6579 (61.1)
65+	335 (22.2)	2004 (18.6)
Region		
North Central	315 (20.9)	2990 (27.8)
Northwest	294 (19.5)	2119 (19.7)
South	717 (47.5)	4058 (37.7)
West	173 (11.5)	1506 (14.0)
Insurance plan		
PPO	843 (55.8)	5993 (55.7)
HMO	153 (10.1)	1105 (10.3)
Comprehensive	158 (10.5)	1101 (10.2)
POS	132 (8.7)	764 (7.1)
Other	206 (13.6)	1692 (15.7)
Charlson Comorbidity Index score		
2	864 (57.2)	6522 (60.6)
3–6	505 (33.4)	3522 (32.7)
>6	141 (9.3)	724 (6.7)
Overweight/Obesity	239 (15.8)	1742 (16.2)
Tobacco use	269 (17.8)	1911 (17.7)
Alcohol abuse	25 (1.7)	175 (1.6)
Drug abuse	7 (0.5)	44 (0.4)
Mental health disorder	98 (6.5)	671 (6.2)
Year		
2013	302 (20.0)	1551 (14.4)
2014	444 (29.4)	2744 (25.5)
2015	360 (23.8)	2672 (24.8)
2016	282 (18.7)	2593 (24.1)
2017	122 (8.1)	1208 (11.2)
Perioperative opioid use		
Short-acting	1505 (99.7)	10,766 (99.98)
Long-acting	6 (0.4)	6 (0.06)
Hydrocodone	727 (48.1)	4881 (45.3)
Oxycodone	676 (44.8)	4706 (43.7)
Tramadol	77 (5.1)	973 (9.0)
Codeine	68 (4.5)	542 (5.0)
Hydromorphone	32 (2.1)	113 (1.0)
Total OME, median (IQR), mg	225 (150–300)	225 (150–300)
Daily OME, median (IQR), mg	45 (30–60)	45 (30–60)
Prescribed days, median (IQR)	5 (4–7)	5 (4–7)

HMO = health maintenance organization; IQR = interquartile range; OME = oral morphine equivalent; ORP = open radical prostatectomy; POS = point of service; PPO = preferred provider organization; RARP = robot-assisted radical prostatectomy.

There were no differences between the two groups with respect to perioperative opioid use. The median total dose of all opioid prescriptions filled during the perioperative period (30 days before admission to 14 days after discharge) was 225 mg OMEs with a median of 5 prescribed days for both groups. Hydrocodone, oxycodone, and tramadol were the most commonly prescribed opioids; less than 1% of patients were prescribed long-acting opioids (Table 2).

**Table 2.** Persistent and Prolonged Opioid Use

	ORP (*n* = 1510),* n *(%)	RARP (*n* = 10,768),* n *(%)
New persistent use (90–180 days)		
Persistent opioid use	146 (9.7)	700 (6.5)
Short-acting	145 (9.6)	697 (6.47)
Long-acting	5 (0.3)	10 (0.1)
Hydrocodone	85 (5.6)	407 (3.8)
Oxycodone	43 (2.8)	175 (1.6)
Tramadol	22 (1.5)	114 (1.1)
Codeine	11 (0.7)	57 (0.5)
Total OME among users, median (IQR), mg	184 (100–405)	200 (113–375)
Daily OME among users, median (IQR), mg	30 (23–45)	33 (23–50)
Prescribed days among users, median (IQR)	5 (3–14)	5 (3–10)
Sensitivity analysis: prolonged use (180–365 days)		
Patients with ≥365 days of follow-up	1194 (79.1)	8274 (76.8)
Prolonged opioid use	41 (3.4)	180 (2.2)

Among patients filling opioid prescriptions perioperatively, 846 (6.9%) patients became new persistent opioid users. In the ORP group, 146 (9.7%) patients filled 1 or more additional opioid prescriptions between 90 and 180 days after discharge *vs* 700 (6.5%) patients in the RARP group (Table 2). Among new persistent users, the median daily dose of opioid prescriptions filled after the perioperative period was slightly higher in RARP compared with ORP patients (33 *vs* 30 OMEs/day) (Table 2).

After adjusting for demographic and clinical covariates, RARP patients were 35% less likely to develop new persistent opioid use compared with those who underwent ORP (aOR 0.65; 95% CI 0.54, 0.79) (Table 3). Patients with a CCI score of 3 to 6 (aOR 1.34; 95% CI 1.16, 1.56), tobacco use (aOR 1.30; 95% CI 1.09, 1.54), and those who resided outside of the northwestern region of the United States (north central 1.62 [1.27, 2.06]; south 1.79 [1.43, 2.24]; and west 1.79 [1.37, 2.35]) had a significantly increased risk of new persistent opioid use.

**Table 3.** Multivariate Logistic Regression Model for New Persistent Opioid Use (90–180 Days)

Characteristics	Adjusted OR (95% CI)
Surgical approach	
ORP	1 [reference]
RARP	0.65 (0.54, 0.79)^a^
Age, years	
55–65	1 [reference]
18–54	1.08 (0.90, 1.29)
65+	0.90 (0.73, 1.11)
Region	
Northwest	1 [reference]
North Central	1.62 (1.27, 2.06)^a^
South	1.79 (1.43, 2.24)^a^
West	1.79 (1.37, 2.35)^a^
Insurance plan	
PPO	1 [reference]
HMO	1.10 (0.85, 1.42)
Comprehensive	0.95 (0.75, 1.21)
POS	0.85 (0.64, 1.14)
Other	0.92 (0.75, 1.14)
Charlson Comorbidity Index score	
2	1 [reference]
3–6	1.34 (1.16, 1.56)^a^
>6	1.26 (0.96, 1.66)
Overweight/Obesity (yes *vs* no)	1.18 (0.98, 1.42)
Tobacco use (yes *vs* no)	1.30 (1.09, 1.54)^a^
Alcohol abuse (yes *vs* no)	1.25 (0.77, 2.04)
Drug abuse (yes *vs* no)	1.60 (0.67, 3.81)
Mental health disorder (yes *vs* no)	1.16 (0.88, 1.52)
Year	
2013	1 [reference]
2014	0.94 (0.74, 1.18)
2015	1.09 (0.87, 1.36)
2016	1.07 (0.85, 1.35)
2017	0.89 (0.66, 1.19)

^a^ Statistically significant if the 95% CI did not cross 1.

CI = confidence interval; OR = odds ratio.

In a sensitivity analysis of patients who were continuously enrolled 365 or more days after discharge, prolonged opioid use (opioid prescription fills, both 90 to 180 days and 181 to 365 days postoperatively) occurred in 41 (3.4%) patients who underwent an ORP and 180 (2.2%) who underwent RARP (aOR 0.62; 95% CI 0.44, 0.88) (Supplementary Table S2). Similar risk factors were observed for prolonged opioid use as for persistent use, including higher CCI score (≥3), tobacco use, and residing in regions other than northwestern United States. In addition, alcohol abuse (aOR 2.15; 95% CI 1.06, 4.35) and undergoing radical prostatectomy in the year 2015 (aOR 1.62; 95% CI 1.06, 2.48; year 2013 as referent) were also associated with prolonged use.

In the sensitivity analysis where we conducted PSM to adjust for confounders, 1510 matched patient pairs were identified and the baseline characteristics were comparable between ORP and RARP (with standardized difference <0.1; Supplementary Table S3). The results were consistent with those of the multivariable logistic regression (risk after PSM: ORP 9.7%, RARP 6.4%; OR 0.64, 95% CI 0.49, 0.84; Supplementary Table S4).

## Discussion

We report opioid use patterns in a cohort of 12,278 patients undergoing either ORP or RARP from July 2013 through June 2017. Among opioid-naïve patients, 84.3% filled a prescription for opioid pain medications perioperatively. The rate of persistent use among patients who filled an opioid prescription in the perioperative setting was 6.9%, similar to rates reported in the literature.^3^ Prolonged use (>180 days) developed in 2.3% of patients who filled a prescription perioperatively, consistent with rates seen in population studies of major thoracic and abdominal surgeries,^9^ as well as plastic and reconstructive surgeries.^8^ RARP was associated with a significantly lower overall risk of developing either persistent or prolonged opioid use when compared with ORP despite having the same quantity of opioid prescribed perioperatively (225 mg of OMEs in the period from 30 days preoperatively through 14 days postoperatively).

Additional risk factors for persistent or prolonged opioid use included higher CCI scores, tobacco use, alcohol abuse, and living in the south, west, or north central region of the United States. The CCI, which aims to predict the 10-year mortality rate for patients with a range of comorbid conditions, functions as a surrogate for overall health. Opioid-naïve patients with a CCI score greater than 3 had a significantly higher risk of persistent and prolonged opioid use after prostatectomy, with the highest risk for prolonged use in patients with a CCI score greater than 6. An increased risk of long-term opioid use in patients with a greater number of comorbid conditions has been previously demonstrated in surgical patients.^8,9^ Likewise, a history of substance abuse is a known risk factor for chronic opioid use postoperatively, and we demonstrate that this pattern persists in patients undergoing prostatectomy. Geographic variation in opioid use may reflect socioeconomic factors or prescription monitoring systems and requires further investigation. Risk reduction strategies and identification of patients at increased risk for opioid dependency are important in reducing opioid use.

Opioid-sparing pathways in the postoperative setting are gaining traction given their ability to maintain patient satisfaction and adequate pain control. The feasibility of reducing or eliminating postoperative opioid prescriptions is reinforced by evidence that patients utilize a small portion of prescribed pills. In a prospective cohort study evaluating opioid utilization after prostatectomy, Patel and colleagues^11^ showed that a median OME dose of 225 mg was prescribed to patients during the 30-day postoperative period (which is consistent with our findings) and only 25% of prescribed pills were taken in both the open and robotic prostatectomy groups and the majority of patients required less than half of the prescribed OMEs. Similarly, Theisen and Davies^12^ demonstrated that 60% of prescribed opioids went unused in patients undergoing open or robotic nephrectomy or prostatectomy, with similar prescribing patterns between open and robotic groups. Efforts are ongoing to introduce and evaluate opioid-reducing and opioid-sparing pathways in urologic surgery. In our cohort, the median OME prescribed in the postoperative period was 225 mg for both open and robot-assisted prostatectomy groups, suggesting that overprescribing is likely a broad problem. Few patients dispose of opioid medications appropriately and diversion of prescribed medication contributes to abuse.^17^ This supports the need for increased stewardship in prescribing as well as prescriber education on postoperative pain management.

Given the national opioid epidemic, efforts to reduce the risk of persistent use and addiction are critical.^2^ The surgical approach may influence postoperative opioid use. Compared with minimally invasive approaches, patients undergoing open colorectal surgery are 18% more likely to use opioids in the immediate postoperative period and use significantly higher daily and total quantities.^18^ Similarly, laparoscopic inguinal hernia repair results in less immediate postoperative pain than open repair.^19^ Robot-assisted prostatectomy has previously been demonstrated to result in lower pain scores in the early postoperative period compared with open surgery.^14^ While there are similar initial opioid fill rates in robot-assisted and open prostatectomy, Kowalczyk et al. demonstrated that fewer patients undergoing RARP required refills within 90 days of surgery.^10^ We expanded on this work and found that patients undergoing RARP are significantly less likely to develop persistent or prolonged opioid use despite similar initial opioid prescription quantity.

Our study is limited by its retrospective nature and the use of insurance claims and is thus subject to potentially inaccurate or incomplete coding. In 2015, there was a transition from ICD-9 to ICD-10 for coding. The code transition may have affected the study cohort and covariates defined by ICD codes nondifferentially and could partly explain the higher rates of persistent use we observed in 2015. Although we can determine the opioid type and quantity filled, the reasons for opioid prescription and amount consumed, if any, by the patient are unknown. Therefore, whether persistent and prolonged opioid use was causally related to persistent postoperative pain, opioid dependence and addiction, or other unrelated conditions, such as a new inciting event, remains unclear. Additionally, we do not have data regarding intraoperative and inpatient opioid use. We did not compare the incidence of opioid dependence after prostatectomy with similar patients undergoing no procedures; however, prior work has shown the rate of developing dependence in the general population to be 0.4%.^4^ Finally, our study relied on a large, population-based claims dataset of insured adults and their dependents with continuous insurance coverage, which may not be generalizable to primary Medicare patients or uninsured populations.

Despite these limitations, we report the largest cohort study to date examining the incidence of persistent opioid medication use after radical prostatectomy in the United States. We specifically included only patients who filled opioid prescriptions during the perioperative period and thus the study population represents the transition of opioid-naive patients from short-term postoperative use to long-term persistent use after surgery. Our population-based study includes outcomes from a range of surgeons and settings, which may more accurately reflect real-world clinical practice than single-surgeon or single-institution reports. Finally, we identify additional risk factors to consider when prescribing opioid medication in the perioperative setting, including the number of medical comorbidities, tobacco use, and history of alcohol abuse.

## Conclusions

Overall, 6.9% of opioid-naive radical prostatectomy patients continued to fill opioid prescriptions 90 days after surgery. Patients who underwent a robot-assisted *vs* open approach were 35% less likely to develop persistent opioid use. Further efforts are needed to develop postoperative opioid prescription protocols to reduce prolonged opioid use among patients undergoing radical prostatectomy.

## Supplementary Material

Supplemental data

Supplemental data

Supplemental data

## Abbreviations Used

aOR: adjusted odds ratio
CCI: Charlson Comorbidity Index
CI: confidence interval
CPT: Current Procedural Terminology
HIPAA: Health Insurance Portability and Accountability Act
HMO: health maintenance organization
ICD-9: *International Classification of Diseases and Related Health Problems, Ninth Revision*
ICD-10: *International Classification of Diseases and Related Health Problems, 10th Revision*
IQR: interquartile range
OME: oral morphine equivalent
OR: odds ratio
ORP: open radical prostatectomy
POS: point of service
PPO: preferred provider organization
PSM: propensity score matches
RARP: robot-assisted radical prostatectomy
