# Supplementary material for: Robot-Assisted Radical Prostatectomy Associated with Decreased Persistent Postoperative Opioid Use
Source: J Endourol. 2020 Apr 16;34(4):475–81. doi: 10.1089/end.2019.0788 (PMC7194325; doi:10.1089/end.2019.0788)
Supplement: Supplemental data [file Supp_Table_S1.pdf]

Supplementary Data

Supplementary Table S1. Diagnosis and Procedure Codes Used to Determine Eligibility

Diagnosis	
ICD-9	
185	Malignant neoplasm of prostate
198.82	Secondary malignant neoplasm of genital organs
233.4	Carcinoma *in situ* of prostate
ICD-10	
C61	Malignant neoplasm of prostate
C79.82	Secondary malignant neoplasm of genital organs
D07.5	Carcinoma *in situ* of prostate
Procedure	
ICD-9	
60.3	Suprapubic prostatectomy
60.4	Retropubic prostatectomy
60.5	Radical prostatectomy
60.69	Other prostatectomy
ICD-10	
0VT00ZZ	Resection of prostate, open approach
0VT04ZZ	Resection of prostate, percutaneous endoscopic approach
0VT07ZZ	Resection of prostate, through natural or artificial opening
0VT08ZZ	Resection of prostate, through natural or artificial opening endoscopic approach
CPT	
55801	Prostatectomy, perineal, subtotal (including control of postoperative bleeding, vasectomy, meatotomy, urethral calibration and/or dilation, and internal urethrotomy)
55810	Prostatectomy, perineal radical
55812	Prostatectomy, perineal radical; with lymph node biopsy(s) (limited pelvic lymphadenectomy)
55815	Prostatectomy, perineal radical; with bilateral pelvic lymphadenectomy, including external iliac, hypogastric, and obturator nodes
55821	Prostatectomy (including control of postoperative bleeding, vasectomy, meatotomy, urethral calibration and/or dilation, and internal urethrotomy); suprapubic, subtotal, one or two stages
55831	Prostatectomy (including control of postoperative bleeding, vasectomy, meatotomy, urethral calibration and/or dilation, and internal urethrotomy); retropubic, subtotal
55840	Prostatectomy, retropubic radical, with or without nerve sparing
55842	Prostatectomy, retropubic radical, with or without nerve sparing; with lymph node biopsy(s) (limited pelvic lymphadenectomy)
55845	Prostatectomy, retropubic radical, with or without nerve sparing; with bilateral pelvic lymphadenectomy, including external iliac, hypogastric, and obturator nodes
55866	Laparoscopy, surgical prostatectomy, retropubic radical, including nerve sparing; includes robotic assistance, when performed
Robotic	
ICD-9	
17.41	Open robot-assisted procedure
17.42	Laparoscopic robot-assisted procedure
17.43	Percutaneous robot-assisted procedure
17.44	Endoscopic robot-assisted procedure
17.45	Thoracoscopic robot-assisted procedure
17.49	Other and unspecified robot-assisted procedure
ICD-10	
8E090CZ	Robot-assisted procedure of the head and neck region, open approach
8E093CZ	Robot-assisted procedure of the head and neck region, percutaneous approach
8E094CZ	Robot-assisted procedure of the head and neck region, percutaneous endoscopic approach
8E097CZ	Robot-assisted procedure of the head and neck region, through natural or artificial opening
8E098CZ	Robot-assisted procedure of the head and neck region, through natural or artificial opening endoscopic approach
8E09XCZ	Robot-assisted procedure of the head and neck region
8E0W0CZ	Robot-assisted procedure of the trunk region, open approach
8E0W3CZ	Robot-assisted procedure of the trunk region, percutaneous approach
8E0W4CZ	Robot-assisted procedure of the trunk region, percutaneous endoscopic approach

(*continued*)

Supplementary Table S1. (Continued)

8E0W7CZ	Robot-assisted procedure of the trunk region, through natural or artificial opening
8E0W8CZ	Robot-assisted procedure of the trunk region, through natural or artificial opening endoscopic approach
8E0WXCZ	Robot-assisted procedure of the trunk region
8E0X0CZ	Robot-assisted procedure of upper extremity, open approach
8E0X3CZ	Robot-assisted procedure of upper extremity, percutaneous approach
8E0X4CZ	Robot-assisted procedure of upper extremity, percutaneous endoscopic approach
8E0XXCZ	Robot-assisted procedure of upper extremity
8E0Y0CZ	Robot-assisted procedure of lower extremity, open approach
8E0Y3CZ	Robot-assisted procedure of lower extremity, percutaneous approach
8E0Y4CZ	Robot-assisted procedure of lower extremity, percutaneous endoscopic approach
8E0YXCZ	Robot-assisted procedure of lower extremity
CPT/HCPCS	
55866	Laparoscopy, surgical prostatectomy, retropubic radical, including nerve sparing; includes robotic assistance, when performed
S2900	Surgical techniques requiring use of a robotic surgical system (list separately in addition to code for the primary procedure)
