# Supplementary material for: Robot-Assisted Radical Prostatectomy Associated with Decreased Persistent Postoperative Opioid Use
Source: J Endourol. 2020 Apr 16;34(4):475–81. doi: 10.1089/end.2019.0788 (PMC7194325; doi:10.1089/end.2019.0788)
Supplement: Supplemental data [file Supp_Table_S2-S3.pdf]

Supplementary Table S2. Mutivariate Logistic Regression Model for Prolonged Opioid Use (90–180 + 180–365 Days)

Characteristics	*Adjusted OR (95% CI)*
Surgical approach	
ORP	1 [reference]
RARP	0.62 (0.44, 0.88)[a]
Age, years	
55–65	1 [reference]
18–54	0.81 (0.56, 1.17)
65+	0.95 (0.65, 1.40)
Region	
Northwest	1 [reference]
North Central	2.05 (1.25, 3.34)[a]
South	2.08 (1.31, 3.30)[a]
West	2.32 (1.36, 3.96)[a]
Insurance plan	
PPO	1 [reference]
HMO	1.16 (0.73, 1.86)
Comprehensive	1.23 (0.80, 1.90)
POS	1.18 (0.69, 2.02)
Other	1.33 (0.91, 1.96)
Charlson Comorbidity Index score	
2	1 [reference]
3–6	1.70 (1.28, 2.27)[a]
>6	1.97 (1.24, 3.13)[a]
Overweight/Obesity (yes *vs* no)	0.88 (0.60, 1.28)
Tobacco use (yes *vs* no)	1.51 (1.10, 2.06)[a]
Alcohol abuse (yes *vs* no)	2.15 (1.06, 4.35)[a]
Drug abuse (yes *vs* no)	0.66 (0.09, 4.90)
Mental health disorder (yes *vs* no)	1.49 (0.93, 2.37)
Year	
2013	1 [reference]
2014	1.24 (0.80, 1.94)
2015	1.62 (1.06, 2.48)[a]
2016	1.15 (0.73, 1.82)
2017	NA

[a]Statistically significant if the 95% CI did not cross one.

CI = confidence interval; HMO = health maintenance organization; OR = odds ratio; ORP = open radical prostatectomy; POS = point of service; PPO = preferred provider organization; RARP = robot-assisted radical prostatectomy.

Supplementary Table S3. Baseline Characteristics After Propensity Score Matching

	ORP (n = *1510), n (%)*	*RARP* (n = *1510), n (%)*	*Standardized difference*
Age, years			0.027
18–54	261 (17.3)	253 (16.8)	
55–65	914 (60.5)	934 (61.9)	
65+	335 (22.2)	323 (21.4)	
Region			0.045
North Central	315 (20.9)	339 (22.5)	
Northwest	294 (19.5)	278 (18.4)	
South	717 (47.5)	705 (46.7)	
West	173 (11.5)	178 (11.8)	
Insurance plan			0.021
PPO	843 (55.8)	855 (56.6)	
HMO	153 (10.1)	156 (10.3)	
Comprehensive	158 (10.5)	154 (10.2)	
POS	132 (8.7)	128 (8.5)	
Other	206 (13.6)	200 (13.2)	
Charlson Comorbidity Index score			0.009
2	864 (57.2)	858 (56.8)	
3 to 6	505 (33.4)	508 (33.6)	
>6	141 (9.3)	144 (9.5)	
Overweight/ Obesity	239 (15.8)	250 (16.6)	0.02
Tobacco use	269 (17.8)	260 (17.2)	−0.016
Alcohol abuse	25 (1.7)	17 (1.1)	−0.045
Drug abuse	7 (0.5)	6 (0.4)	−0.01
Mental health disorder	98 (6.5)	76 (5.0)	−0.063
Year			0.039
2013	302 (20.0)	320 (21.2)	
2014	444 (29.4)	446 (29.5)	
2015	360 (23.8)	360 (23.8)	
2016	282 (18.7)	264 (17.5)	
2017	122 (8.1)	120 (7.9)	

Standardized difference: values <0.1 assumed to indicate negligible difference.
