# Supplementary material for: Robot-Assisted Radical Prostatectomy Associated with Decreased Persistent Postoperative Opioid Use
Source: J Endourol. 2020 Apr 16;34(4):475–81. doi: 10.1089/end.2019.0788 (PMC7194325; doi:10.1089/end.2019.0788)
Supplement: Supplemental data [file Supp_Table_S4.pdf]

Supplementary Table S4. Persistent Opioid Use in Propensity Score-Matched Cohorts

Surgical approach	N *(%)*	*Adjusted OR (95% CI)*
ORP ($N = 1510$)	146 (9.7)	1 [reference]
RARP ($N = 1510$)	97 (6.4)	0.64 (0.49, 0.84)
